# You Look Familiar: How Malaysian Chinese Recognize Faces

**DOI:** 10.1371/journal.pone.0029714

**Published:** 2012-01-11

**Authors:** Chrystalle B. Y. Tan, Ian D. Stephen, Ross Whitehead, Elizabeth Sheppard

**Affiliations:** 1 School of Psychology, University of Nottingham Malaysia Campus, Selangor, Malaysia; 2 School of Psychology, University of St Andrews, St Andrews, Scotland, United Kingdom; University of Utah, United States of America

## Abstract

East Asian and white Western observers employ different eye movement strategies for a variety of visual processing tasks, including face processing. Recent eye tracking studies on face recognition found that East Asians tend to integrate information holistically by focusing on the nose while white Westerners perceive faces featurally by moving between the eyes and mouth. The current study examines the eye movement strategy that Malaysian Chinese participants employ when recognizing East Asian, white Western, and African faces. Rather than adopting the Eastern or Western fixation pattern, Malaysian Chinese participants use a mixed strategy by focusing on the eyes and nose more than the mouth. The combination of Eastern and Western strategies proved advantageous in participants' ability to recognize East Asian and white Western faces, suggesting that individuals learn to use fixation patterns that are optimized for recognizing the faces with which they are more familiar.

## Introduction

Studies examining cultural differences in perceptual tasks, such as scene perception [1], [2] and susceptibility to visual illusions [3], [4], have found that East Asian observers tend to adopt a global perceptual style by integrating salient objects and background context while white Western observers show a more local style by preferentially attending to focal objects rather than configural information.

Recent studies on face recognition found similar cross-cultural differences in the eye movement strategies that observers use when perceiving faces. Human faces provide vital information about individuals' identities and characteristics (including gender, age, health, and attractiveness). Although faces are similar, in that they comprise the same basic features in approximately the same configuration, they are easily distinguished by observers. Evidence from neuroimaging studies suggests that there is a specialized mental module, possibly located in the fusiform face area [5], dedicated to face processing. This ability to recognize different faces proficiently may have social and evolutionary advantages, including allowing us to remember specific individuals' behavior in social situations and recognizing cooperators and defectors [6], [7]. Thus, investigating how people extract visual information in relation to their ability to recognize faces is of particular interest for human social behavior [8]. It is known that people are better at recognizing own- than other-race faces [9], possibly because of greater familiarity [10]. However, only recently have researchers asked whether the same underlying attentional strategies are deployed. Systematic cross-cultural differences were found in the fixation patterns of East Asian and white Western observers [11], [12]. More specifically, it has been suggested that East Asians' tendency to focus on the center of the face, in the nose area, indicated a configural style of processing, while white Western observers' triangular looking pattern (moving between the eyes and mouth) may indicate more local processing of individual features [11].

A further study suggested that British-born Chinese observers show either Eastern or Western eye movement strategies, with fixations landing predominantly around either the eyes or nose region [13]. British-born Chinese participants were equally accurate in recognizing East Asian and white Western faces, suggesting that increased familiarity with other-race faces enhances recognition abilities. However, our examination of the data suggests that most participants actually employed intermediate looking strategies, with none of the 20 participants showing a bias of over 10% towards either strategy. Further, the bias values are normally distributed (Kolmogorov-Smirnov test p≥0.2) around a mean bias of just 2.5% towards an Eastern strategy, not bimodally distributed as would be expected if participants could be validly classified into either Western or Eastern strategists. Kelly et al. [13] acknowledge that the strategies represent an influence of both cultures. This may indicate that looking strategies are learnt in order to best recognize the faces that are visible during development.

The current study aimed to investigate whether exposure and familiarity with Western culture affects Malaysian Chinese participants' recognition accuracy and eye movement strategies by requiring participants to perform a face recognition task on East Asian, white Western, and African faces. Although Malaysia is an East Asian country, it is strongly multicultural and influenced by Western culture. Malaysian FM radio stations are composed of 40% Malay language stations, 26% English, and 15% Chinese [14]. 86% of movies shown in Malaysian cinemas are Western and a mere 14% are local. This compares to 57% local movies in Chinese and Japanese cinemas [15]. The ethnic composition of Malaysia is also highly diverse, with Malays composing 50.4% of the population, Chinese 23.7%, indigenous 11%, Indian 7.1%, and others composing 7.8% (including white Westerners and Africans) [16]. Data from the Department of Immigration in 2006 reported that 8.6% of the country's 19,444 expatriates originated from the United Kingdom, placing it as the fourth largest source of expatriates to Malaysia. Conversely, no African (or black majority) country was listed as a major source of immigrants in any class of international migration [17]. Given the diverse nature of the country, we predicted that Malaysian Chinese students enrolled in a branch campus of a British university would be equally good at recognizing East Asian and white Western faces, but not the less familiar African faces. Participants were also predicted to use a mixture of East Asian and white Western eye movement strategies to perceive faces.

## Methods

### Participants

Twenty-two East Asian young adults (10 males, 12 females, mean age 21.86 years) participated in this study. All participants were Malaysian Chinese students attending the University of Nottingham Malaysia Campus, and have not lived outside of Malaysia for more than three years. All participants had normal or corrected vision and were given a bar of chocolate for their participation. Written informed consent was obtained from all participants and the protocol was approved by the University of Nottingham, School of Psychology Ethics Committee.

### Materials

Stimuli consisted of 60 images distributed equally among race (East Asian, white Western, and African) and sex, photographed in a lighting booth painted with Munsell N5 neutral gray paint and illuminated with d65 fluorescent tubes, in high-frequency fixtures to reduce the effects of flicker (Verivide, UK). The East Asian and white Western stimuli were obtained from a set of images collected at the University of St Andrews, UK while the African stimuli were collected from the University of Pretoria, South Africa. All images were color calibrated after Stephen et al. [18]. The images, which were 270×333 pixels in size, were presented at a distance of 60 cm on gray background using a 17in TFT monitor with a screen resolution of 1280×1024pixels. The images were aligned on the eyes' position and cropped around the face using Psycho-Morph software [19]. The background was edited using Adobe Photoshop CS. Presentation of the stimuli was controlled by the Tobii Studio software.

### Eye Tracking

Eye movements were recorded with an on-screen remote eye tracking system (Tobii T60), in which an infrared camera is integrated to the lower part of the 17in TFT monitor. The eye tracker performs binocular tracking at a data sampling rate of 60 Hz and has high accuracy (0.5°) and drift compensation (less than 0.3°). Each task began with a calibration procedure as implemented in the Tobii Studio software to ensure accurate tracking of eye gaze.

### Procedure

The experiment involved two phases: the learning phase and the face recognition phase. During the learning phase, 30 faces (5 male, 5 female East Asian, 5 male, 5 female white Western, and 5 male, 5 female African) were shown on a Tobii eye tracker and participants were asked to rate the faces for attractiveness on a seven-point Likert scale. Participants then filled out a questionnaire to distract them from remembering the faces. Upon completion of the questionnaire, participants performed a face recognition task in which 60 faces (10 male, 10 female East Asian, 10 male, 10 female white Western, and 10 male, 10 female African, of which half were new faces) were presented. Participants gave a yes or no response to indicate if they had seen the face before.

On each trial, a central fixation cross was presented for one second followed by a face presented pseudorandomly in one of four quadrants of the computer screen to avoid fixation bias. The face stimulus was presented for 5 seconds in both phases and was followed by a question that required a response in relation to the task (e.g. a forced-choice question as to whether the participant had seen the face before). Each response was subsequently followed by the central fixation cross, which preceded the next face stimulus.

### Data Analysis

A' values were calculated to determine participants' recognition accuracy. A', which is a non-parametric equivalent of d', indexes participants' sensitivity to old and new faces taking into account both hits (i.e. correct detection of an old face) and false alarms (i.e. incorrect identification of a new face as an old face).

**Figure 1:**
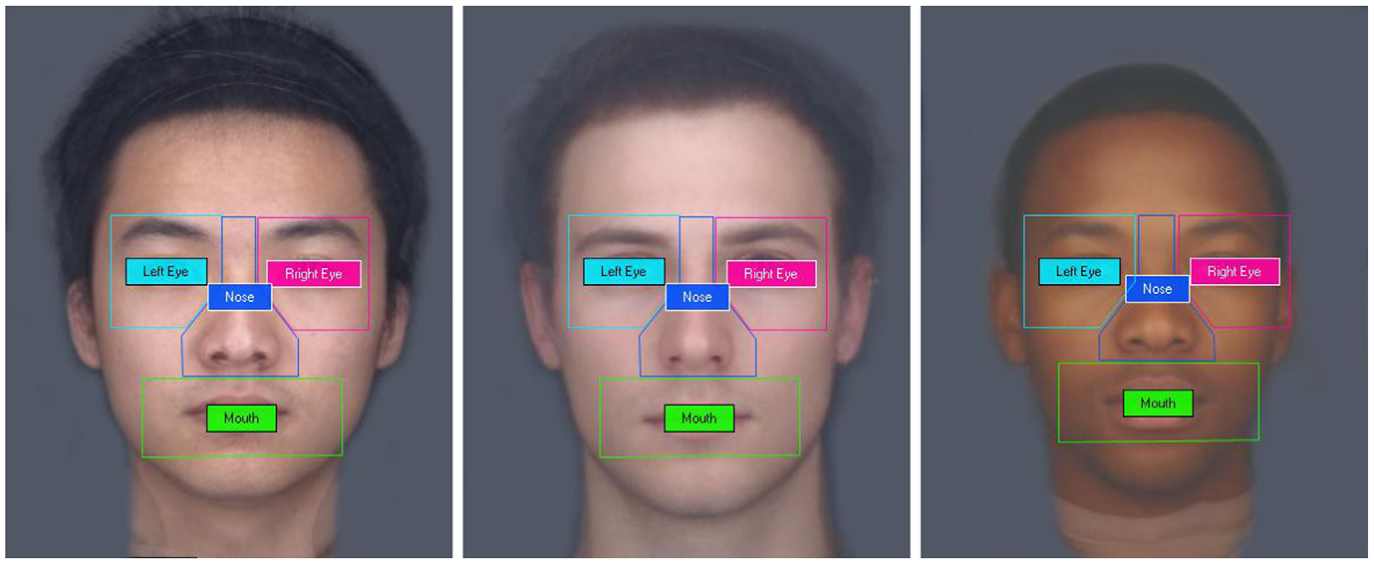
The predefined area of interest (AOI) used to analyze eye gaze. Composite images are shown for illustration purposes. Real faces were used in the actual experiment.

The data was processed directly from the eye tracker using the Tobii Studio software. The eye tracker samples at 60 Hz (approximately every 17 milliseconds). Fixation, which is the main measurement used in this study, is defined by the standard Tobii fixation filter as two or more consecutive samples falling within a 35 pixel radius. The total number of fixations a participant made within the predefined areas (eyes, mouth, and nose) was accumulated using the area of interest (AOI) analysis, see Figure 1. Each participant completed 60 trials for 5 seconds each. To ensure the validity of the eye tracking data, only participants who had an average number of fixations equal to or more than 5 counts were included in the data set. Two participants (1 male) did not meet this criterion because of poor calibration and were excluded from the eye tracking analysis. Both of these participants fixated for less than 0.8 seconds per 5-second trial and only 13% of gaze sample was collected by the eye tracker.

## Results

### Recognition Accuracy

**Figure 2:**
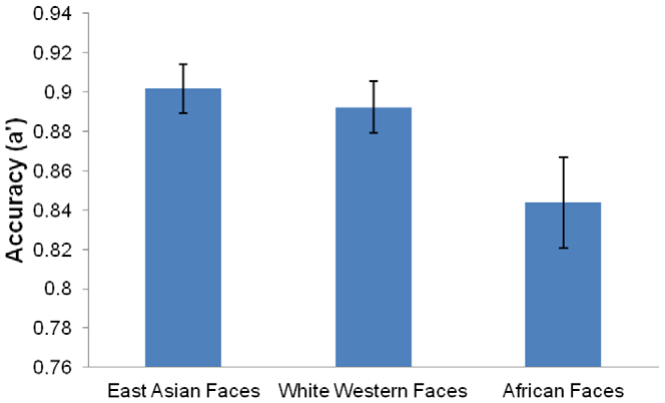
Malaysian Chinese participants' recognition accuracy for East Asian, white Western, and African faces. Error bars report standard errors of the mean. Participants recognized East Asian faces significantly better than African faces.

A' values were not normally distributed. Hence, data was reflected and transformed using a square root function (i.e. the square root of one minus A') before analysis. However, the bar graph below (Figure 2) represents untransformed A' values for easier interpretation.

A one-way repeated measures ANOVA showed that there was a significant effect of race on participants' recognition accuracy (a'), *F*(2, 42) = 3.96, *p*<.05. Post-hoc tests using LSD revealed that participants were significantly better at recognizing East Asian than African faces (*p*<.05). However, recognition accuracy for East Asian faces was not significantly different from white Western faces (*p* = .51). Results also indicated a trend towards white Western faces being recognized more accurately than African faces (*p* = .08), see Figure 2.

### Fixation Counts

The mean number of fixations over each 5 second trial during the face recognition task was 9.43.

**Figure 3:**
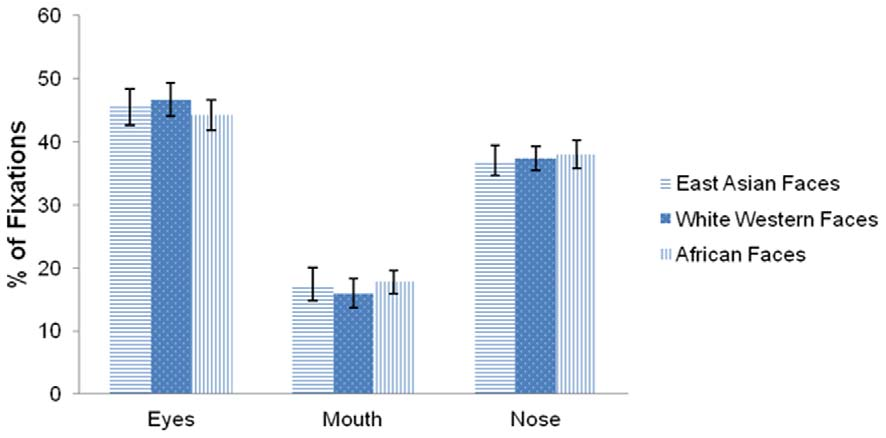
Percentages of fixations landing on the eyes, mouth, and nose during the face recognition task. Error bars report standard errors of mean.

A 3 (Race of Face: East Asian, white Western, or African)×3 (Feature of Face: Eyes, Mouth, or Nose) ANOVA was conducted on the percentage of all fixations falling on the eyes, nose, and mouth for each participant. There was a main effect of Feature only, *F*(2, 38) = 27.88, *p*<.001, indicating that participants use similar fixation patterns to process faces regardless of the race of the face, and do not adjust their looking strategies according to the race of specific faces. Post-hoc tests using LSD revealed that participants looked at the eyes (*p*<.001) and nose (*p*<.001) significantly more than the mouth. The percentage of fixations landing on the eyes and nose did not differ significantly, although there was a trend towards a greater percentage falling on the eyes (*p* = .06; Figure 3). This suggests that participants are using an intermediate between Eastern and Western recognition strategies to recognize faces.

## Discussion

The current study examined Malaysian Chinese participants' fixation patterns and recognition accuracy for East Asian, white Western, and African faces. The primary findings were that (a) Malaysian Chinese participants performed equally well at recognizing East Asian and white Western faces, but less well at recognizing African faces, and (b) when perceiving all three races of faces, Malaysian Chinese participants displayed a combination of Eastern and Western eye movement strategies with fixations clustered around the eyes and nose more than the mouth. These findings suggest that Malaysian Chinese participants learn to use a fixation pattern that is advantageous for recognizing the multicultural faces that they encounter.

As compared with the Chinese or Japanese population, who are largely influenced by their respective cultures, Malaysian Chinese individuals have greater exposure to the West, as reflected in the high percentage of English radio stations and Western movies shown in Malaysian cinema [14], [15]. Our participants all also attend a branch campus of a British university, increasing their exposure to Western people and culture. Familiarity with Western culture may have influenced the fixation pattern that Malaysian Chinese individuals employ so that they recognize most accurately the faces that they most frequently encounter. Rather than adopting the Eastern or Western fixation pattern, Malaysian Chinese participants employed a mixed strategy that is strikingly different from either of these fixation patterns previously reported [11], but perhaps more aligned with that of British-born Chinese individuals [13]. Of particular note in this study was the low percentage of fixations directed at the mouth, which rendered the eye movement patterns of Malaysian Chinese observers distinct from both the East Asian and white Western participants in previous studies [11], [12]. The combination of Eastern and Western strategies proved advantageous for Malaysian Chinese participants as they accurately recognized East Asian and white Western faces.

However, the recognition accuracy for African faces was significantly lower than for East Asian faces, perhaps due to participants' lack of attention to features believed to be of high diagnostic value for African faces (i.e. the lower facial features). Previous studies have shown that people of different races use different facial features to describe [20] and recognize [11] faces. Furthermore, it was found that the other-race effect can be reduced by directing participants' attention to the features thought to have high diagnostic value (and are most frequently mentioned in descriptions) for faces of a given ethnic group (e.g. eyes for white Westerners and lips for Africans) [21]. In line with this finding, our results suggest that attention to the eyes is an effective strategy that enhances Malaysian Chinese participants' ability to recognize white Western faces, but the lack of focus on the mouth was detrimental to participants' ability to recognize African faces.

Finally, our results suggest that the cognitive mechanisms involved in face recognition show plasticity (as predicted by evolutionary theory [22]), learning to use facial cues to identity that have the highest diagnostic value in the local population.
